# Supplementary material for: Synthesis of Bio-Based Thermoset Mixture Composed of Methacrylated Rapeseed Oil and Methacrylated Methyl Lactate: One-Pot Synthesis Using Formed Methacrylic Acid as a Continual Reactant
Source: Polymers (Basel). 2023 Apr 7;15(8):1811. doi: 10.3390/polym15081811 (PMC10146403; doi:10.3390/polym15081811)
Supplement: Supplementary file 1 [file polymers-15-01811-s001.zip › polymers-2254173-supplementary.pdf]

Supplementary material for article: ***Synthesis of bio-based thermoset mixture composed of methacrylated rapeseed oil and methacrylated methyl lactate: One-pot synthesis using formed methacrylic acid as a continual reactant***

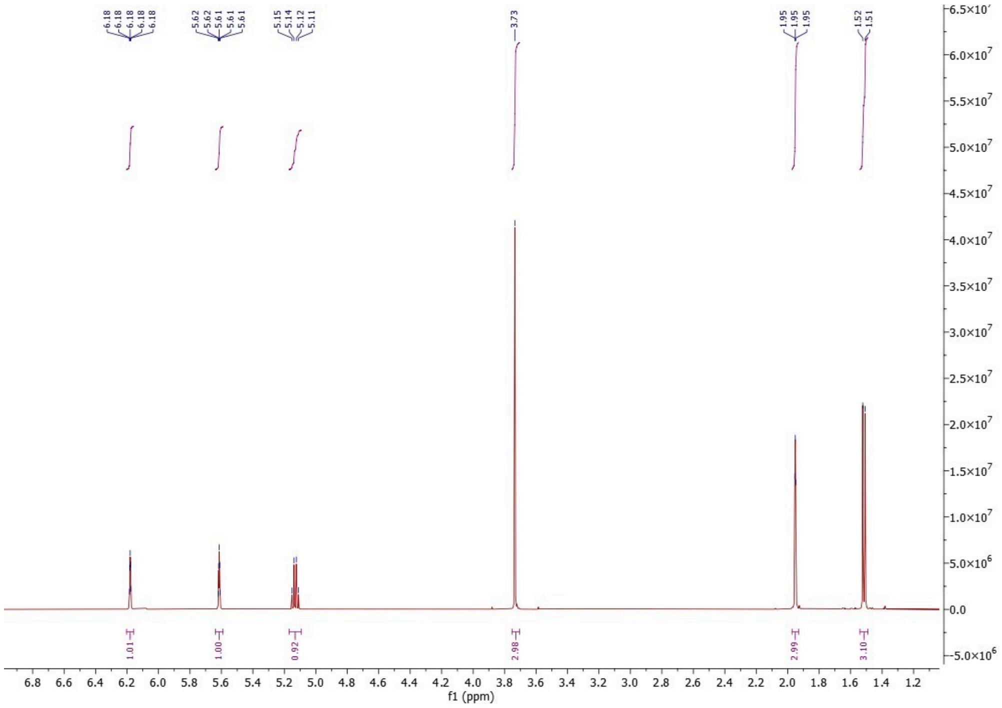

Figure S1. Methacrylated methyl lactate (MeLaMMA):^{1}H NMR (Figure S1) ($CDCl_3$, 500 MHz): δ(ppm) 6.18 (p; *J* = 1.08; 1.08; 1.07; 1.07 Hz; 1H), 5.62–5.61 (p; *J* = 1.63; 1.63; 1.61; 1.61 Hz; 1H), 5.15–5.11 (q; *J* = 7.05; 7.05; 7.05 Hz; 1H), 3.73 (s; 3H), 1.95 (t; *J* = 1.32; 1.32 Hz; 3H), 1.53 (d; *J* = 7.08 Hz; 3H).

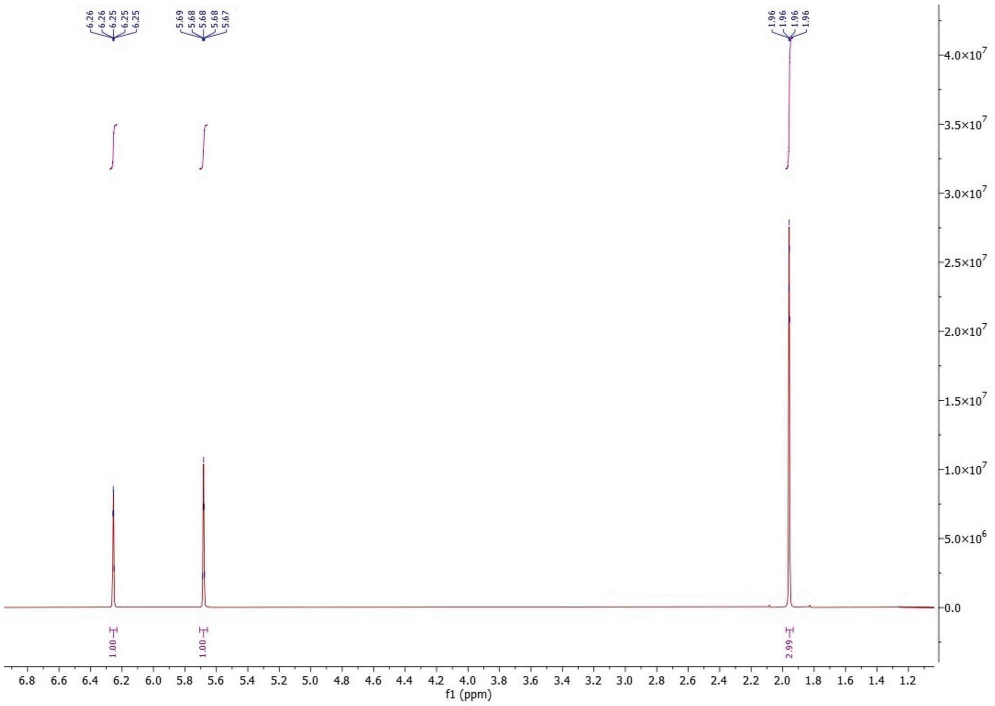

Figure S2. Methacrylic acid (MA):^{1}H NMR (Figure S2) ($CDCl_3$, 500 MHz): δ(ppm) 6.26-6.25 (dd; J = 1.52; 0.95 Hz; 1H), 5.69–5.67 (p; J = 1.66; 1.66; 1.63; 1.63 Hz; 1H), 1.96 (dd; J = 1.63; 1.01 Hz; 3H).

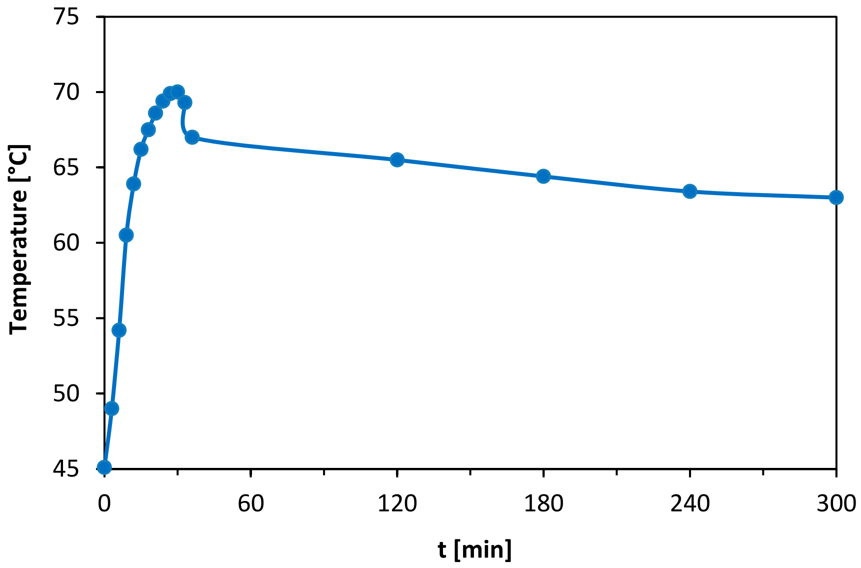

Figure S3. The dependence of the temperature on time during the initial phase of oil's epoxidation.

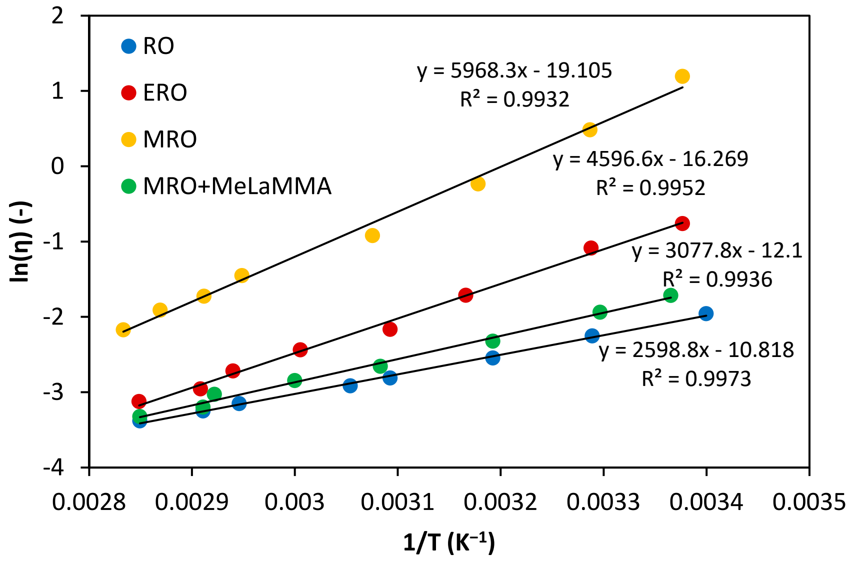

Figure S4. The graphical representation of the Arrhenius equation applied for a dependence of apparent viscosity on the temperature.

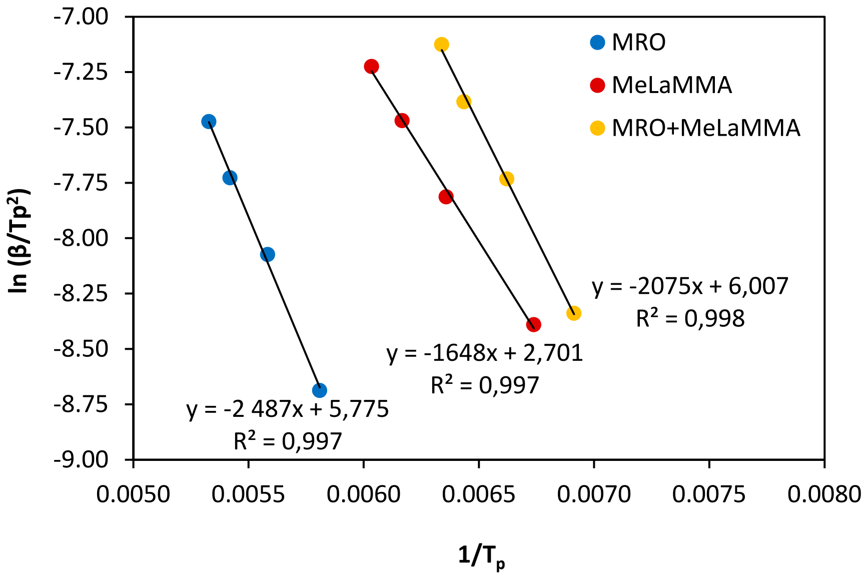

Figure S5. Graphical illustration of Kissinger's theory applied to synthesized resins.

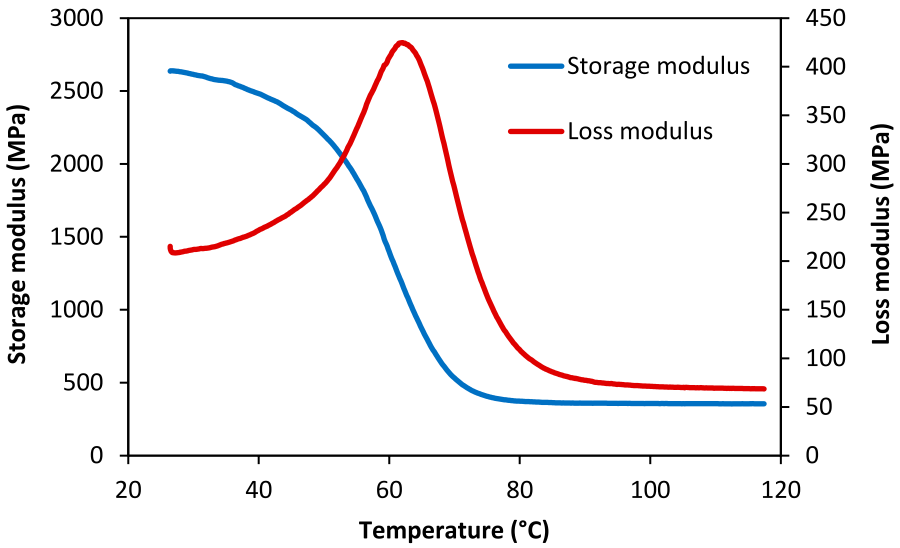

Figure S6. The dependence of the storage modulus and loss modulus of methacrylated methyl lactate (MeLaMMA) on temperature.

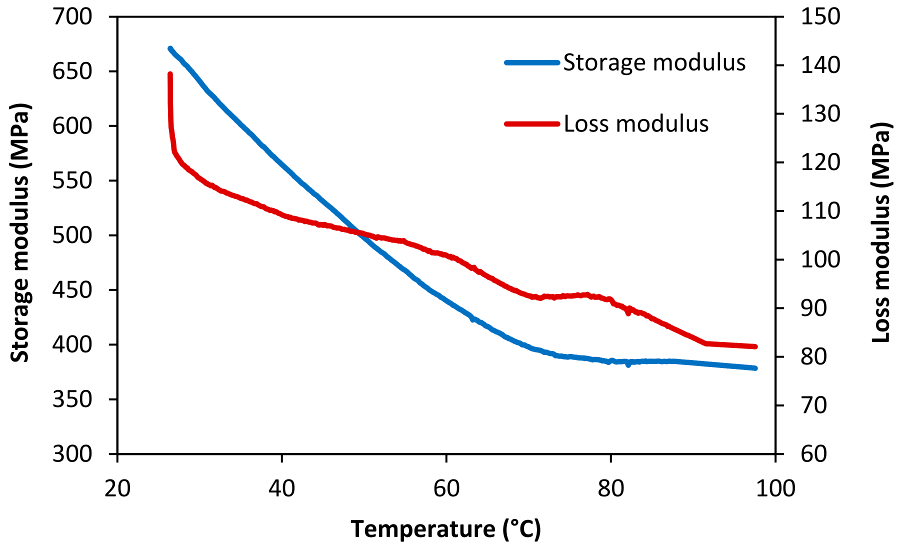

Figure S7. The dependence of the storage modulus and loss modulus of methacrylated rapeseed oil (MeRO) on temperature.

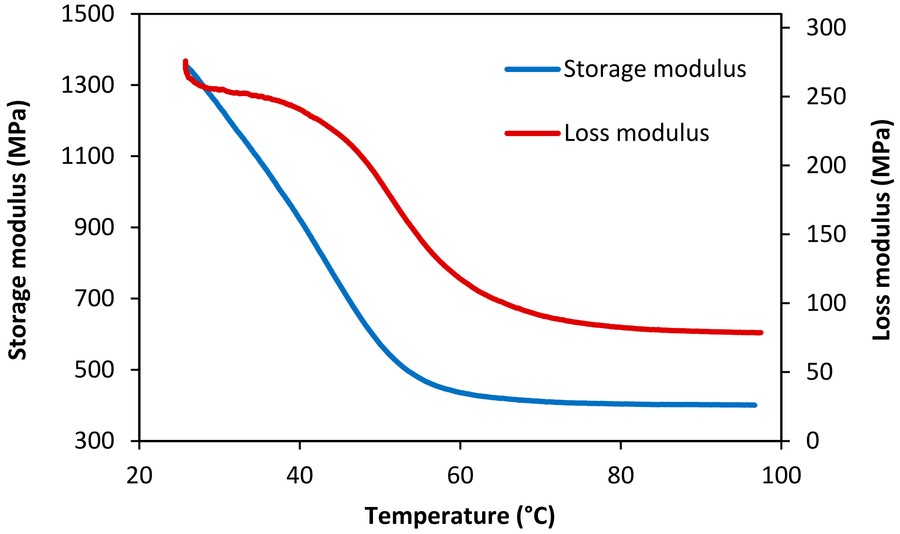

Figure S8. The dependence of the storage modulus and loss modulus of the synthesized thermoset mixture (containing MeLaMMA and MeRO) on temperature.
